# Understanding the Association Between Intolerance of Uncertainty and Problematic Smartphone Use: A Network Analysis

**DOI:** 10.3389/fpsyt.2022.917833

**Published:** 2022-07-11

**Authors:** Chang Liu, Lei Ren, Kuiliang Li, Wei Yang, Ye Li, Kristian Rotaru, Xinyi Wei, Murat Yücel, Lucy Albertella

**Affiliations:** ^1^BrainPark, Turner Institute for Brain and Mental Health and School of Psychological Sciences, Monash University, Clayton, VIC, Australia; ^2^Military Medical Psychology School, Fourth Military Medical University, Xi'an, China; ^3^School of Psychology, Army Medical University, Chongqing, China; ^4^Psychological Counseling Center, Xijing University, Xi'an, China; ^5^Department of Accounting, Monash Business School, Monash University, Caulfield, VIC, Australia; ^6^Department of Psychology, Renmin University of China, Beijing, China

**Keywords:** intolerance of uncertainty, problematic smartphone use, network analysis, central symptoms, bridge symptoms

## Abstract

**Background:**

Existing research has demonstrated that intolerance of uncertainty (IU) is associated with problematic smartphone use (PSU). However, little is known about how different IU components such as uncertainty-related beliefs, emotions, and behaviors may impact on different PSU symptoms.

**Methods:**

Extending previous research, the current study examined the specific associations between IU components and PSU symptoms via a symptom-level network approach. A regularized partial correlation network consisting of different IU components and PSU symptoms was estimated among 1,849 Chinese university students. We examined pathways and influential nodes (i.e. central components/symptoms and bridge components/symptoms) within the IU-PSU network.

**Results:**

The strongest pathway linking IU and PSU was between emotional reactions to uncertainty and coping-motivated smartphone use. Importantly, emotional reactions toward not having enough information (a reflection of emotional reactions to uncertainty) may act as both a central and a bridge component in maintaining the whole IU-PSU network.

**Conclusions:**

The results are in line with the I-PACE model and highlight that PSU may be a coping response for negative emotions derived from uncertainty. Finally, the current findings highlight the potential of interventions targeting intolerance of uncertainty for reducing PSU.

## Introduction

Problematic smartphone use (PSU), characterized by excessive smartphone use and associated negative daily-life consequences (1), has received increasing attention from the psychology/psychiatry field in recent years. Young adults, especially university students, may be more likely to experience PSU due to digital nativity (2). The median prevalence of PSU among children and young people was 23.3% (3). Importantly, PSU is often associated with physical and psychological concerns, including depression, anxiety, sleep impairments, and spinal disorders (4–6). Given the high prevalence and negative consequences associated with PSU, there is a call to understand the mechanism underlying PSU (2, 7). The Interaction of Person-Affect Cognition-Execution (I-PACE) theoretical model proposes that personal predispositions (e.g., personality traits, affective and cognitive responses) may serve as vulnerability factors and contribute to the development and maintenance of PSU (8, 9). In line with this model, which features such core components as predisposing variables, emerging research has attempted to explore how psychopathology constructs may underpin PSU.

One candidate transdiagnostic risk factor for PSU is intolerance of uncertainty (IU) (10). IU, which is defined as the tendency to react negatively to uncertain situations and events due to negative beliefs about uncertainty and its implications (11), has been examined in relation to anxiety-related and obsessive-compulsive disorders (12, 13). Emerging research found that IU may be associated with problematic behaviors including problematic alcohol use, problematic internet use, problematic gambling, compulsive buying and disordered eating (14–16). To date, only one study examined the relationship between IU and PSU. Using a repeated-measures design, the study found that baseline IU level showed a significant positive correlation with PSU measured after 1 month (10). According to the I-PACE model, the relationship between IU and PSU can be explained as follows: individuals with certain predisposing traits (e.g., IU) may be more likely to experience excessive worry and somatic stress when facing ambiguous situations. These negative emotional reactions may diminish top-down control, increasing engagements in behaviors that may provide instant, short-term relief (e.g., spending a large amount of time on their mobile phones, searching for reassurance). This process may be enhanced through negative reinforcement, with stress-reduction reinforcing smartphone use such that the individual consequently will engage in smartphone use whenever facing the negative emotional state induced by uncertainty.

Despite significant contributions from previous research, there is a lack of a nuanced understanding of how distinct IU components may be associated with specific PSU symptoms. PSU and IU have been examined as unitary constructs in previous research (10). However, PSU may manifest as a heterogeneous condition involving different symptoms (e.g., loss of control, disruption, withdrawal, preoccupation and tolerance), with each symptom distinct from one another in terms of its relative importance (17, 18). Similarly, IU has been identified as a multidimensional construct consisting of uncertainty-related beliefs, emotions, and behaviors (19). These different IU components may play different roles in the development and maintenance of specific PSU symptoms. Neglecting the construct/symptomatic heterogeneity may be problematic as it may hinder a more a nuanced understanding of the relationships existing between different components of IU to individual PSU symptoms (20). This drawback has been demonstrated by several recent studies. For instance, individual PSU symptoms have shown different associations with self-control (21), problematic internet use (22) and neuroticism (23). Meanwhile, different components of IU were found to have distinct effects on generalized anxiety disorder (24). In viewing this drawback, it has been proposed that psychopathology research may benefit from moving beyond disorder-level analysis to a more fine-grained symptom-level analysis (25, 26).

One statistical method which is suitable for the symptom-level analysis is the network approach. Theoretically, the network framework conceptualizes psychopathology as a complex system with nodes (both symptom and non-symptom variables) that interact and reinforce each other via their potential causal linkage (i.e., edges). This is in line with the I-PACE model, which proposes that PSU arises from interactions between predisposing variables, affective and cognitive responses to stimuli, and executive functions (e.g., inhibitory control). As a symptom-oriented graphical approach, the network approach offers the ability to visualize the complex relationships between psychological constructs at a more granular level (e.g. at the level of components and symptoms). Within a network, variables (both symptom and non-symptom) are depicted as nodes, which may connect (via edges) and reinforce each other and constitute mental health conditions (27). By observing the network structure and examining the centrality index, the researcher may get an insight of the symptoms that share a closer connection and thus form a cluster, the symptoms that are more influential than others in terms of their effects, the symptoms that may have cross-cluster connections, as well as the pathways linking different symptoms (28–30). Such insights may not only help researchers elucidate the mechanisms that underlie the component-to-symptom associations, but also shed light on developing more precise and targeted interventions (31).

To our knowledge, no study to date has examined how individual IU components may contribute to specific PSU symptoms. To address this gap and extend previous research on IU-PSU association, the current study modeled the component-to-symptom relationships between IU and PSU via the network approach. Specifically, we examined: (1) the unique associations between IU components and PSU symptoms, (2) the most influential nodes that maintain the network system, and (3) the most influential nodes that bridge the IU cluster and the PSU cluster. Based on existing theory research (15), we hypothesized that emotional reactions toward uncertainty may bridge to coping-motivated smartphone use. As compulsivity (i.e., continued use despite problems) is a cardinal feature of addiction (32), we hypothesized that the symptom characterized by compulsivity may be the central PSU symptom. Further, based on previous research, we hypothesized that the “preference for planning ahead” may be the central IU component (19).

## Methods

### Ethics Statement

The data collection procedure followed the Declaration of Helsinki and was approved by the Ethics Committee of the First Affiliated Hospital of the Fourth Military Medical University (Project No. KY20202063-F-2).

### Participants

Participants were undergraduate students from five universities (i.e. Xijing University, Yan'an University, Xi'an International Studies University, Xi'an Shiyou University and Shangluo University) in Shaanxi Province, China. The data was collected via a Chinese online survey platform (Wenjuanxing). Before the survey, a WeChat (one of the largest instant messaging application in China) message with links to the online survey was sent to all students who were currently enrolled in the aforementioned universities. All participants provided informed consent before participation. Demographic information was collected at the start of the survey. All questions in the survey were set as forced responses (i.e., participants need to provide responses to all questions before they can submit). Therefore, there were no incomplete responses. One hundred and seventy-six participants were excluded due to failing the two honesty check items (e.g., participants did not choose the second option when they responded to “Please choose the second option for this question”). The final sample consisted of 1,849 participants (59% female, mean age = 19.00, SD = 1.32, range = 17–23 years).

### Measures

#### Problematic Smartphone Use

Problematic smartphone use during the past year was measured by a modified version of the Chinese translated nine-item Internet Gaming Disorder Scale - Short Form (33) This is the only scale that adopted the Diagnostic and Statistical Manual of Mental Disorders (fifth edition) (34) criteria for Internet Gaming Disorder and comprehensively addressed nine symptoms (i.e., preoccupation, withdrawal, tolerance, loss of control, giving up other activities, continuing despite problems, deception, escapism/avoidance, and negative consequences) that characterize addictive behaviors. The modified version (with “gaming” replaced by “smartphone use”) has been used to measure PSU in several existing studies, with good internal consistency (35–37). Sample items include “Do you feel more irritability, anxiety or even sadness when you try to either reduce or stop your smartphone use” and “Have you continued your smartphone use despite knowing it was causing problems between you and other people.” Participants responded on a 5-point Likert-type scale ranging from 1 = “never” to 5 = “very often.” The scale demonstrated good internal consistency in the current study (McDonald's omega = 0.89).

#### Chinese Version of the Intolerance of Uncertainty Scale-Short Form (C-IUS-12)

The C-IUS-12 was used to measure different components of IU (38). Participants reported to what degree each item applies to them on a 5-point Likert-type scale ranging from 1 = “not at all characteristic of me” to 5 = “entirely characteristic of me.” Sample items include “Unforeseen events upset me greatly” and “When I am uncertain I can't function very well.” The scale demonstrated good internal consistency in the current study (McDonald's omega = 0.87).

### Data Analysis

Graphical Gaussian model (GGM) was used to estimate the IU-PSU network (39) GGM is undirected network, and its edge represents the pairwise relations between nodes after controlling for all other nodes in the network. The network was estimated on the basis of nonparametric Spearman's Rho correlations (40). Spearman correlations are recommended when data are skewed (41–43). Graphical Least Absolute Shrinkage and Selection Operator (LASSO) algorithm was used for regularization. By purposefully introducing a penalty hyperparameter, trivially small correlations were shrunk to zero during the regularization process. This helps reduce false-positive relationships and obtain a sparse network (40). The hyperparameter value may range from 0 (resulting in a more sensitive network with more remaining edges) to 1 (resulting in a more specific network with less remaining edges). To ensure that edges remaining in the final network are genuine, the hyperparameter was set to 0.5 to balance the tradeoff between sensitivity and specificity (40).

Two clusters were pre-defined before analysis, namely the PSU symptom cluster (items from the PSU scale) and the IU cluster (items from the C-IUS-12 scale). The resulting network consisted of nodes (individual items from the PSU scale and C-IUS-12 scale) and edges (regularized partial correlation between nodes) (40). The Fruchterman-Reingold algorithm was used for visualizing the layout (44). Within the network, nodes that are strongly correlated were placed next to each other, while the least connected nodes were placed further apart. Blue edges represent positive correlations, and red edges represent negative correlations. The edge thickness represents the magnitudes of the regularized partial correlation between nodes. These procedures were conducted via the R package qgraph (45).

To identify the central nodes, we calculated the node expected influence (the sum of edge weight of a given node) using the qgraph package. Compared to traditional centrality indices (i.e., strength, closeness, and betweenness), expected influence accounted for both positive and negative edges and was demonstrated to be a better approach for identifying influential nodes with the presence of negative edges (46). Further, a recent study (47) showed that only node expected influence successfully predicted how strongly changes in nodes were related to change in the remainder of the nodes when compared with other centrality measures (i.e., strength and predictability). Given that the presented network contained both positive and negative edges, it was more appropriate to use node expected influence than other centrality indices. Central nodes are considered as core to understanding the etiology and intervention targets of mental health concerns due to their strong interconnectedness within the network (46). To identify important nodes that bridge the IU-PSU connection, we calculated the bridge expected influence (i.e. the sum of edge weights from a given node to the other cluster) (48). Nodes with higher bridge expected influence are considered to play a more central role in activating nodes from the opposite cluster. The bridge expected influence was calculated via the R package networktools (49). In order to identify the number of central and bridge nodes, we conducted a rigorous method with a blind 85th percentile cutoff on the value of node expected influence and bridge expected influence to avoid the confirmation bias that might arise when we interpret centrality statistics. Consequently, three nodes will be identified as central and three nodes will be identified as bridge nodes.

To ensure network accuracy, we bootstrapped the confidence intervals of the edge weights with 2,000 bootstrapped samples. To ensure stability of the centrality index (i.e. node expected influence and bridge expected influence), we calculated the correlation stability (CS)-coefficient via a bootstrapped case-dropping procedure (with 2,000 bootstrapped samples). In order to be considered stable, the CS-coefficient should not be lower than 0.25 and ideally above 0.50 (50). We also conducted bootstrapped difference tests to examine whether two edge weights or two node centralities (i.e., node expected influence and bridge expected influence) differ significantly from one another. The level of significance was set to *p* < 0.05 for all bootstrapped comparison tests. The aforementioned steps were conducted via the R package bootnet (50).

## Results

### Descriptive Statistics

Table 1 displays the descriptive statistics of examined variables. The sample consisted of 1,849 participants (59% female, mean age = 19.00, SD = 1.32, range = 17–23 years).

**Table 1:** Mean, standard deviation (SD), skewness, and kurtosis of each variable selected in the present networks.

**Items**	***M* (SD)**	**Skewness**	**Kurtosis**
IU 1 (“Unforeseen events upset me greatly”)	3.0 (1.1)	−0.37	−0.90
IU 2 (“It frustrates me not having all the information I need”)	3.0 (1.1)	−0.45	−0.95
IU 3 (“One should always look ahead so as to avoid surprises”)	3.8 (0.9)	−1.09	1.51
IU 4 (“A small, unforeseen event can spoil everything, even with the best of planning”)	3.4 (1.0)	−0.54	−0.40
IU 5 (“I always want to know what the future has in store for me”)	3.5 (1.1)	−0.62	−0.25
IU 6 (“I can't stand being taken by surprise”)	2.9 (1.1)	−0.07	−0.84
IU 7 (“I should be able to organize everything in advance”)	3.3 (0.9)	−0.43	−0.22
IU 8 (“Uncertainty keeps me from living a full life”)	2.6 (1.1)	0.10	−0.95
IU 9 (“When it's time to act, uncertainty paralyzes me”)	2.8 (1.1)	−0.05	−1.09
IU 10 (“When I am uncertain I can't function very well”)	2.8 (1.1)	−0.09	−1.20
IU 11 (“The smallest doubt can stop me from acting”)	2.5 (1.1)	0.22	−1.01
IU 12 (“I must get away from all uncertain situations”)	2.3 (1.1)	0.50	−0.65
IU total	35.9 (8.1)		
PSU 1 (“Do you feel preoccupied with your smartphone use? (Some examples: Do you think about previous smartphone use or anticipate the next smartphone use? Do you think smartphone use has become the dominant activity in your daily life?”))	2.7 (1.0)	0.05	−0.62
PSU 2 (“Do you feel more irritability, anxiety or even sadness when you try to either reduce or stop your smartphone use?”)	1.6 (0.8)	1.32	1.57
PSU 3 (“Do you feel the need to spend increasing amount of time engaged smartphone use in order to achieve satisfaction or pleasure?”)	1.8 (0.9)	1.03	0.92
PSU 4 (“Do you systematically fail when trying to control or cease your smartphone use?”)	2.2 (1.0)	0.53	−0.32
PSU 5 (“Have you lost interest in previous hobbies and other entertainment activities as a result of your engagement with the smartphone?”)	1.6 (0.8)	1.41	1.76
PSU 6 (“Have you continued your smartphone use despite knowing it was causing problems between you and other people?”)	1.8 (1.0)	1.05	0.51
PSU 7 (“Have you deceived any of your family members, therapists or others because of the amount of your smartphone use?”)	1.7 (0.8)	1.13	0.80
PSU 8 (“Do you use your smartphone in order to temporarily escape or relieve a negative mood (e.g., helplessness, guilt, anxiety)?”)	2.2 (1.1)	0.57	−0.54
PSU 9 (“Have you jeopardized or lost an important relationship, job or an educational or career opportunity because of your smartphone use?”)	1.5 (0.8)	1.51	2.22
PSU total	17.0 (6.1)		

*M, mean; SD, standard deviation*.

### Network Structure

**Figure 1:**
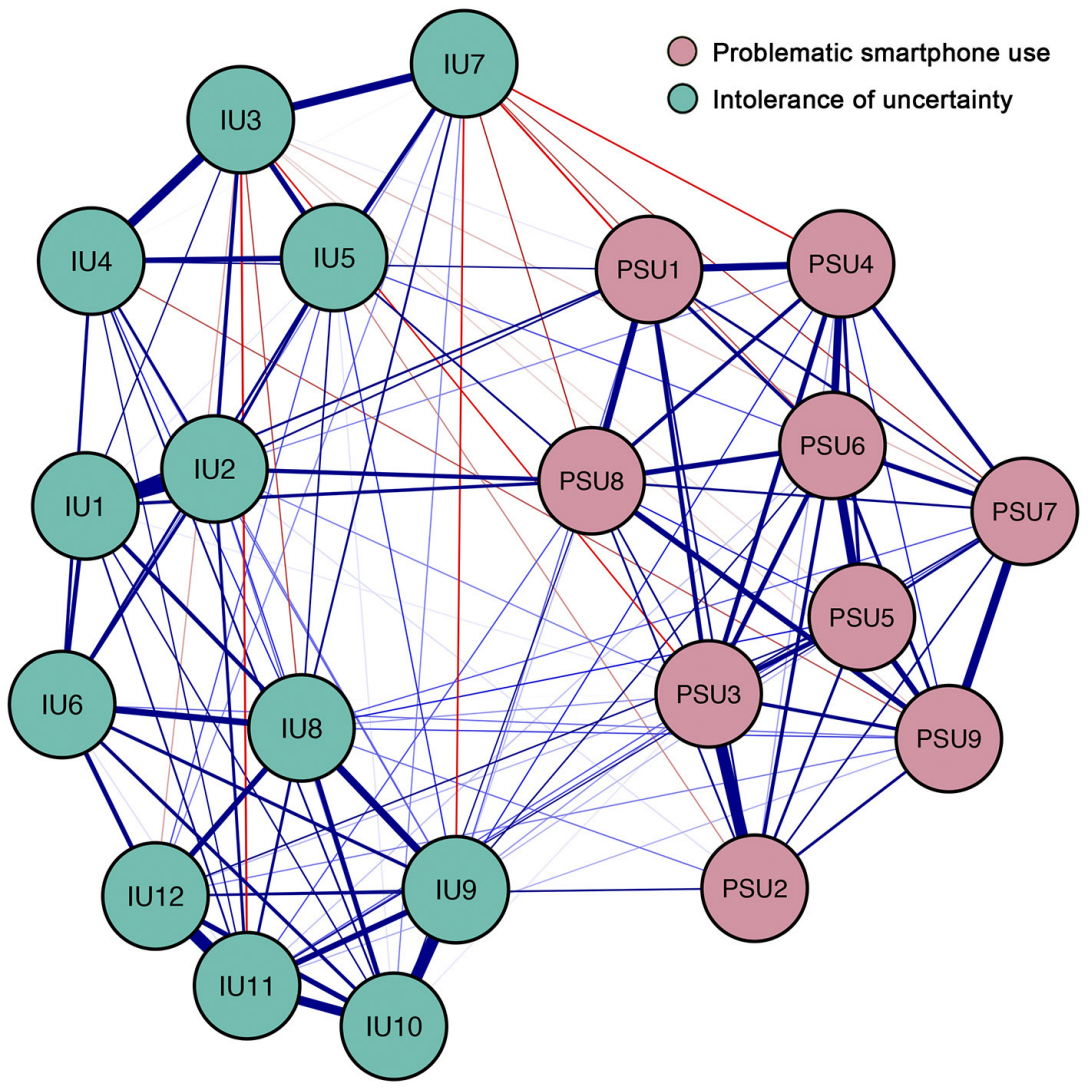
Network structure of different components of intolerance of uncertainty and symptoms of problematic smartphone use. Blue edges represent positive correlations, red edges represent negative correlations. The thickness of the edge reflects the magnitude of the correlation. Cut value = 0.03. The text of variables selected in network can be seen in Table 1.

Figure 1 presents the 21-item IU-PSU network. There are 138 of 210 (66%) possible edges (weight range from -.05 to .43) within the network. Overall, more positive edges (*n* = 123) were observed than negative edges (*n* = 15). The strongest between-cluster edges were between IU 2 (“It frustrates me not having all the information I need”) and PSU 8 (“Do you use your smartphone in order to temporarily escape or relieve a negative mood?”; edge weight = 0.11); IU 1 (“Unforeseen events upset me greatly”) and PSU 8 (“Do you use your smartphone in order to temporarily escape or relieve a negative mood?”; edge weight = 0.08). The strongest negative between-cluster edges were between IU 7 (“I should be able to organize everything in advance”) and PSU 1 (“Do you feel preoccupied with your smartphone use?”; edge weight = −0.05).

Within the PSU symptom cluster, all of the connections between nodes are positive (weight range from 0.01 to 0.31). The strongest edges are between PSU 2 (“Do you feel more irritability, anxiety or even sadness when you try to either reduce or stop your smartphone use?”) and PSU 3 (“Do you feel the need to spend increasing amount of time engaged smartphone use in order to achieve satisfaction or pleasure?”; edge weight = 0.31). Within the IU cluster, most of the connections between nodes are positive (weight range from −0.05 to 0.43). The strongest edges are between IU 1 (“Unforeseen events upset me greatly”) and IU 2 (“It frustrates me not having all the information I need”; edge weight = 0.43). The bootstrapped 95% confidence interval is relatively narrow, indicating that the edges of the IU-PSU network are considered to be accurate (Supplementary Figure S1). Supplementary Figure S2 showed the bootstrapped difference test for edge weights.

### Centrality of Network

**Figure 2:**
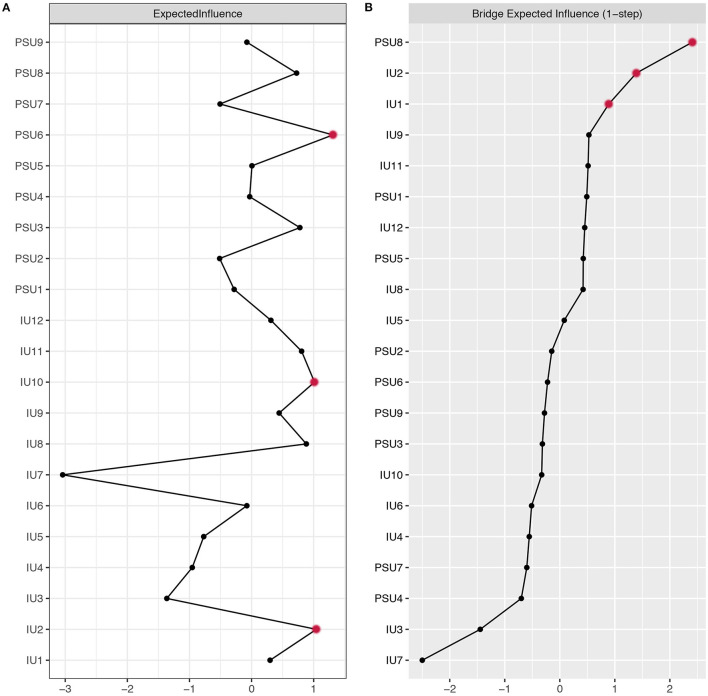
Centrality plot depicting the **(A)** expected influence and **(B)** bridge expected influence of each variable selected in the present network (*z*-score). The text of variables selected in network can be seen in Table 1.

The expected influence values are presented in Figure 2A. PSU 6 (“Have you continued your smartphone use despite knowing it was causing problems between you and other people?”), IU 2 (“It frustrates me not having all the information I need”) and IU 10 (“When I am uncertain I can't function very well”) showed high expected influence and were considered to be central nodes in the IU-PSU network. The CS-coefficient for node expected influence (value = 0.75) was larger than 0.5, indicating this centrality index was adequately stable (Supplementary Figure S3). Supplementary Figure S4 showed the bootstrapped difference test for node expected influence.

### Bridge Nodes

The bridge expected influence values are presented in Figure 2B. Three bridging nodes with the highest bridge expected influence were PSU 8 (“Do you use your smartphone order to temporarily escape or relieve a negative mood?”), IU 2 (“It frustrates me not having all the information I need”) and IU 1 (“Unforeseen events upset me greatly”). The CS-coefficient for bridge expected influence (value = 0.75) was larger than 0.5, indicating this centrality index was adequately stable (Supplementary Figure S5). Supplementary Figure S6 showed the bootstrapped difference test for node bridge expected influence.

## Discussion

Existing research demonstrates that IU may contribute to PSU when measured using item sum scores (10). Employing network analysis, the current study further explored the complex interrelationship between IU components and PSU symptoms. In line with our hypothesis and the I-PACE model, we found that the strongest inter-cluster connections were between negative emotional reactions to uncertainty (IU 1 and IU 2) and coping-motivated smartphone use (PSU 8). Specifically, individuals with higher levels of negative emotional reactions to uncertainty may experience more intense arousal when facing uncertain situations. This may reduce their inhibitory control and predispose them to engage in short-term behaviors (e.g., smartphone use). By constantly pairing stress-reduction with smartphone use behaviors, such behaviors may be acquired as a maladaptive coping strategy when distressed by uncertainty.

Similar to previous studies, we found intra-cluster connections were generally stronger than inter-cluster connections (20, 51). The strongest edges within the IU cluster were between IU 1 (“Unforeseen events upset me greatly”) and IU 2 (“It frustrates me not having all the information I need”). These results are consistent with previous research on the network structure of IU (19, 24). For the PSU cluster, we found PSU 2 (“Do you feel more irritability, anxiety or even sadness when you try to either reduce or stop your smartphone use”) has a strong connection to PSU 3 (“Do you feel the need to spend increasing amount of time engaged smartphone use in order to achieve satisfaction or pleasure?”). Despite tolerance and withdrawal usually going hand in hand, we may not rule out the possibility that such a correlation may be due to different causal sources. This potential confounding influence should be taken into consideration when interpreting the observed relationship between PSU 2 and PSU 3.

Supporting our second hypothesis, the centrality analysis showed that PSU 6 (“Have you continued your smartphone use despite knowing it was causing problems between you and other people?”) was the most central PSU symptom. Previous research has shown that continued use is a core symptom of PSU in Chinese adolescents (44). Our results replicate this finding in a university student sample. Meanwhile, the preference for planning ahead (IU 7) did not emerge as the central IU component as we hypothesized. Previous research showed that the factor structure of the IU measure differed across racial groups (52). Thus, it is unsurprising that the central node may be different from previous research using Italian samples (19). We found IU 2 (“It frustrates me not having all the information I need”), and IU 10 (“When I am uncertain I can't function very well”) were central IU nodes in the current study. Compared to negative beliefs about uncertainty (IU 3, 4, 5, 7), emotional (IU 2) and behavioral (IU 10) reactions to uncertainty may be more critical for maintaining the structure of the IU-PSU network.

In the network presented in our study, node bridge centrality may cast light on the specific role played by different components of IU in the development and maintenance of PSU. In the IU cluster, IU 2 (“It frustrates me not having all the information I need”) and IU 1 (“Unforeseen events upset me greatly”) were identified as bridge nodes for the IU-PSU association. This suggests that IU 2 and IU 1 have stronger associations with symptoms of PSU than other components of IU. Thus, from a network perspective, targeting IU 2 and IU 1 may be more effective at reducing symptoms of PSU than targeting other components of IU. As previously stated, both IU 1 and IU 2 were strongly correlated with coping-motivated smartphone use, indicating a potential pathway for interventions. In the PSU cluster, PSU 8 (“Do you use your smartphone in order to temporarily escape or relieve a negative mood?”) was identified as the bridge node. This indicates that PSU 8 might be susceptible to the IU cluster.

It is worth mentioning the crucial role of IU 2 (i.e. as both a central node and a bridge node) was also reported in a network study that examined the relationship between IU components and generalized anxiety disorder symptoms (24). This indicates that IU 2 may be a target for research and interventions across different mental health conditions, in line with current transdiagnostic models of psychopathology (e.g., RDOC) (53). In fact, a recent study had showed that the centrality of node in the cross-sectional and between-subject networks may be ill-defined and the supporting evidence is inconsistent (47). Thus, this hypothesis needs to be further investigated in future studies.

The current findings have important implications for intervention development. It has been proposed that addressing central nodes may reduce its associated symptoms within the whole network, while addressing bridge nodes may disrupt the illness pathway and reduce the comorbidity/co-occurrence (54, 55). Based on current findings, IU 2, identified as both a central node and a bridge node, may be a potential target for early interventions of PSU. Specifically, information about individuals' tendency to experience negative emotional reactions toward uncertainty may be used to identify individuals at risk of developing PSU and target early interventions to reduce risk for developing PSU. A randomized controlled trial of Intolerance of Uncertainty Treatment demonstrated promising results in reducing IU level and anxiety-related symptoms (56). Theoretically, reducing one's negative emotional responses toward uncertainty (e.g., worry and anxiety) may lead to less motivation for excessive smartphone use. Coping-skills interventions, which aim to help individuals develop adaptive coping responses under distress, may be another candidate intervention. Specifically, individuals may recognize excessive smartphone use as the only means of coping strategy for uncertainty-related distress and be over-reliant on it despite aversive consequences. Thus, helping these individuals recognize and successfully implement other adaptive coping strategies may reduce the reliance on the maladaptive ones (e.g., excessive smartphone use). Indeed, coping-skills interventions have been shown effective in reducing substance misuse (57, 58). Whether such intervention may be effective in reducing PSU symptoms warrant future research.

Several limitations should be considered when interpreting the current results. First, the study utilized a cross-sectional design, which prevented us from drawing conclusions on temporal associations. Future studies should consider addressing this limitation by utilizing longitudinal study designs. Second, the study used a convenient sampling approach among university students. This, in combination with the data-driven nature of the network approach, may reduce the generalizability of the current findings to other samples. Future research is required to replicate our results in more diverse populations (e.g., clinical samples). Third, both IU and PSU were measured by self-report scale, which may induce recall bias. Fourth, the present network investigated between-subject effects at a group level. That is, the network structure of a single individual may not be replicated in the same way. Finally, despite being used in several empirical studies, the validation of the modified nine-item Internet Gaming Disorder Scale - Short Form is still ongoing. Nevertheless, the scale demonstrated good internal consistency in the current study.

## Conclusion

The current study advances the understanding of the relationship between IU and PSU by examining how distinct IU components relate to specific PSU symptoms via a network approach. By highlighting the inter-group connection between emotional reactions to uncertainty and coping-motivated smartphone use, our findings may shed light on future research aiming to develop theoretical understanding and interventions for reducing PSU. Further, as PSU symptoms may fluctuate over time, it is plausible that the network structure may also change in the course of time. Future studies should examine the temporal association between IU components and PSU symptoms to see if the network structure identified in the current study may change over time. Finally, the current findings highlight the potential of IU interventions for reducing symptoms of PSU; future studies are needed to determine this potential.

## Data Availability Statement

The raw data supporting the conclusions of this article may be available from the corresponding author on reasonable requests. Requests to access these datasets should be directed to LR, rl_fmmu@163.com.

## Ethics Statement

The studies involving human participants were reviewed and approved by Ethics Committee of the First Affiliated Hospital of the Fourth Military Medical University. The questionnaire was completed online in the WeChat application after electronic informed consent was obtained.

## Author Contributions

CL, LR, and LA developed study idea and design. CL wrote the original draft of this manuscript. All authors contributed to revising subsequent versions of the paper.

## Funding

LR's involvement in this research was funded by the Fourth Military Medical University (2021JSTS30). MY has received funding from Monash University and Australian Government funding bodies such as the National Health and Medical Research Council (NHMRC; including Fellowship #APP1117188), the Australian Research Council (ARC), the Australian Defence Science and Technology (DST) Group, and the Department of Industry, Innovation and Science (DIIS). He has also received philanthropic donations from the David Winston Turner Endowment Fund, Wilson Foundation, as well as payments in relation to court-, expert witness-, and/or expert review-reports.

## Conflict of Interest

The authors declare that the research was conducted in the absence of any commercial or financial relationships that could be construed as a potential conflict of interest.

## Publisher's Note

All claims expressed in this article are solely those of the authors and do not necessarily represent those of their affiliated organizations, or those of the publisher, the editors and the reviewers. Any product that may be evaluated in this article, or claim that may be made by its manufacturer, is not guaranteed or endorsed by the publisher.
